# Successful radical surgical resection of initially unresectable intrahepatic cholangiocarcinoma by downsizing chemotherapy with gemcitabine plus cisplatin: a case report

**DOI:** 10.1186/s40792-017-0395-y

**Published:** 2017-11-21

**Authors:** Ryosuke Takayanagi, Shigetsugu Takano, Kensuke Sugiura, Hideyuki Yoshitomi, Katsunori Furukawa, Tsukasa Takayashiki, Satoshi Kuboki, Atsushi Kato, Masaru Miyazaki, Masayuki Ohtsuka

**Affiliations:** 1 Department of General Surgery, Chiba University Graduate School of Medicine, 1-8-1 Inohana, Cyuou-ku, Chiba, 260-8677 Japan; 2 Department of Gastroenterological Surgery, Mita Hospital, International University of Health and Welfare, Tokyo, Japan

**Keywords:** Intrahepatic cholangiocarcinoma (ICC), Biliary tract cancer (BTC), Initially unresectable BTC, Downsizing chemotherapy, Conversion surgery

## Abstract

**Background:**

Intrahepatic cholangiocarcinoma (ICC) is a subtype of biliary tract cancer (BTC). Recently, downsizing chemotherapy has been applied to initially unresectable BTCs, including ICC.

**Case presentation:**

We report a case of liver resection in a 23-year-old woman who was diagnosed with initially unresectable ICC attached to the inferior vena cava, with portal vein (PV) cavernous transformation. Positron emission tomography (PET) showed fluorodeoxyglucose (FDG) uptake in the para-aortic lymph nodes. Upon using downsizing chemotherapy (the combination of gemcitabine [GEM] and cisplatin [CDDP]), the size of tumor reduced by 55% and FDG uptake in the para-aortic lymph node metastases disappeared. A right hemihepatectomy was performed, along with dissection of lymph nodes, including the para-aortic lymph nodes. The PV cavernous transformation was preserved to maintain collateral flow as much as possible, as it was considered to originate from a congenital anomaly. Pathological examination revealed that R0 resection was performed and that there were no viable neoplastic cells remaining in the para-aortic lymph nodes. The patient is alive at 31 months after initial treatment, with a local recurrence.

**Conclusion:**

Downsizing chemotherapy with GEM plus CDDP followed by radical surgical resection is an attractive treatment for initially unresectable BTC.

## Background

Intrahepatic cholangiocarcinoma (ICC), a form of biliary tract cancer (BTC), is an uncommon cancer arising from the epithelium of the intrahepatic bile duct. Surgical resection is the only curative treatment option that leads to long-term survival in BTC patients. However, the majority of patients are initially diagnosed with unresectable tumors due to locally advanced or metastatic disease. Recently, systemic chemotherapy has been utilized, and some initially unresectable BTCs can become resectable following downsizing chemotherapy. Herein, we describe a case of successful radical surgical resection following downsizing chemotherapy for initially unresectable advanced ICC.

## Case presentation

**Fig. 1:**
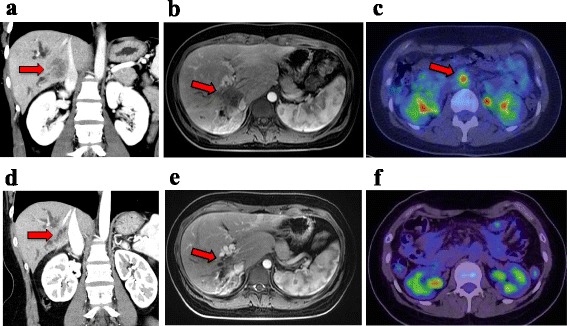
Radiological examinations before (**a**, **b**, and **c**) and after (**d**, **e**, and **f**) downsizing chemotherapy. **a**, **b** Computed tomography (CT) and magnetic resonance imaging (MRI) images of the liver showed focal dilation of the right intrahepatic bile duct and a 42-mm tumor in hepatic segments 1 to 8; the tumor was attached to the inferior vena cava (IVC) (**a** axial view of CT, **b** coronal view of MRI). **c** Positron emission tomography (PET) showed fluorodeoxyglucose (FDG) uptake in the para-aortic lymph nodes. **d**, **e** The size of tumor decreased to 18 mm, and the tumor was no longer attached to the IVC (**d** axial view of CT, **e** coronal view of MRI). **f** No FDG uptake was seen in the para-aortic lymph nodes

A 23-year-old woman with no relevant past medical or surgical history was referred from a local hospital after complaining of right upper quadrant pain for several weeks. On physical examination, right upper quadrant tenderness was the only abnormal finding during the first visit. Initial laboratory findings were also unremarkable, except for a high level of carbohydrate antigen 19-9 (CA19-9; 20,300 U/ml). Abdominal computed tomography (CT) and magnetic resonance imaging (MRI) revealed focal dilation of the right intrahepatic bile duct and a low contract effect tumor measuring 42 mm in hepatic segments 1 and 8; ICC was suspected (Fig. 1a, b). The tumor was attached to the inferior vena cava (IVC) and had invaded the right portal vein (PV) leading to cavernous transformation instead of canonical PV. Positron emission tomography (PET) showed fluorodeoxyglucose (FDG) uptake in the primary liver tumor and para-aortic lymph nodes (Fig. 1c). We consider the cases those in which surgical resection could not be achieved even by aggressive surgical procedures, including combined vascular resection as unresectable ICCs. On the basis of these findings, the patient was diagnosed with initially unresectable ICC with para-aortic lymph node metastasis and subsequently treated with a combined chemotherapy regimen of gemcitabine (GEM 1000 mg/m^2^) and cisplatin (CDDP 25 mg/m^2^). This combination was intravenously administered on days 1 and 8 and was repeated every 3 weeks under a downsizing regimen.

**Fig. 2:**
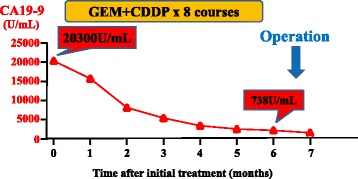
Serum carbohydrate antigen 19-9 (CA19-9) levels during downsizing chemotherapy

**Fig. 3:**
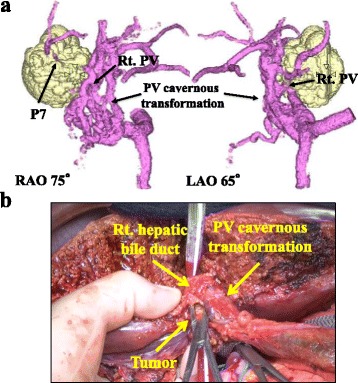
**a** Three-dimensional figures presenting the relation of tumor location and portal vein (PV) cavernous transformation before downsizing chemotherapy with two different angles using SYNAPSE VINCENT® (FUJIFILM, Co., Ltd., Tokyo, Japan). **b** Intraoperative image showing liver resection. Intraoperative findings revealed that liver duodenum ligament was soft and the PV cavernous transformation was distant from the tumor

After eight courses of combination chemotherapy over 5 months, CT, MRI, and PET imaging demonstrated an effective response to chemotherapy. The size of the primary tumor had decreased to 18 mm (55% reduction), and the tumor was no longer attached to the IVC (Fig. 1d, e). FDG uptake in the para-aortic lymph nodes almost disappeared (Fig. 1f), and the level of CA19-9 decreased to 738 U/ml (Fig. 2). The effect of downsizing chemotherapy was partial response (PR) in RECIST criteria. The TNM staging of pre- and post-chemotherapy were T3N1M1 stage IVb and T1N0M0 stage I in UICC criteria, respectively. Nevertheless, the PV cavernous transformation did not improve. We thus concluded that this PV cavernous transformation had originated from a congenital anomaly (Fig. 3a). After discussion with the patient and her family, we decided to perform radical surgical resection to achieve the cure of disease. Right hemihepatectomy and dissection of lymph nodes, including the para-aortic lymph nodes, were performed. During right PV resection, the PV cavernous transformation was preserved to maintain collateral flow through the PV as much as possible (Fig. 3b). The margins of the right bile duct showed no evidence of malignancy during intraoperative frozen section analysis.

**Fig. 4:**
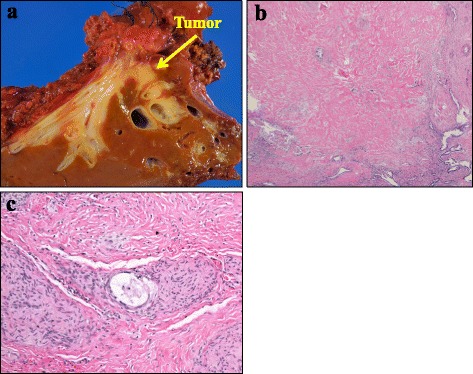
Pathological findings. **a** Macroscopic findings. **b** Microscopic findings of a surgical specimen stained with hematoxylin and eosin demonstrating that more than 50% of the tumor area was replaced with fibrosis (Evans criteria IIb). **c** Microscopic findings of the extracapsular invasion in the para-aortic nerve

Microscopic pathological examination showed that R0 (no residual tumor) resection had been successful and that more than 50% of the tumor cells had been replaced with fibrosis (Evans’ criteria IIb) (Fig. 4a, b). No viable tumor cells were visible in the para-aortic lymph nodes that showed FDG uptake in the initial PET scan. However, there was slight extracapsular invasion in the para-aortic nerve (Fig. 4c). According to the pathological findings (well differentiated tubular adenocarcinoma, pT3, pN0, pM1 (OTH, para-aortic nerve)), UICC stage of this patient was defined as stage IVb. The patient suffered from bile duct stricture as a postoperative complication. After re-suture of the stump of the bile duct with enough patency in reoperation, the patient made a satisfactory recovery and was discharged on postoperative day 11. The patient is alive 31 months after the initial treatment (24 months after operation) with a local tumor recurrence.

### Discussion

In surgical resection for ICC, R0 resection is one of the most favorable prognostic factors [1, 2]. The prognosis of R1 and R2 (microscopic and macroscopic residual tumor) resection is comparable to the prognosis of patients who do not undergo surgical resection and those who receive only palliative treatment [3]. Furthermore, the number of lymph node metastases is another important prognostic factor in ICC. Patients with more than three lymph nodes metastases have more unfavorable outcomes than patients with one or two lymph node metastases (3-year survival: 0 vs. 50%, respectively) [3].

Many patients are diagnosed initially with unresectable tumors due to locally advanced or metastatic disease, silent clinical symptoms, involvement of blood vessels, extension into both hepatic lobes, or rapid disease progression [4]. The optimal treatment for patients with locally advanced unresectable or metastatic disease is yet to be determined. Recently, systemic chemotherapy has contributed to improvements in overall survival. In the UK, a phase III randomized controlled trial demonstrated that the median survival time (MST) of overall survival (OS) with GEM plus CDDP combination therapy was significantly improved compared to that with gemcitabine alone (11.7 vs. 8.3 months, respectively, *p* < 0.001) [5]. For unresectable BTC, GEM plus CDDP combination therapy is recommended as a standard therapy.

A previous report looked at the effectiveness of downsizing chemotherapy (distinguished from neoadjuvant chemotherapy) and subsequent so-called “conversion surgery” for initially unresectable BTC [6]. We also recently reported that in 10 of 39 (25.6%) locally advanced unresectable BTC patients, the size of the tumor was reduced by downsizing chemotherapy, and conversion surgery was successfully performed as a result. Additionally, this downsizing chemotherapy and conversion surgery strategy led to longer survival than that with chemotherapy alone (MST: 17.9 months for chemotherapy plus surgical resection vs. 12.4 months for chemotherapy alone) [7]. Although there is a certain bias in patient selection, this could represent a promising treatment for initially unresectable locally advanced BTC.

The efficacy of neoadjuvant chemotherapy against potentially resectable BTC is uncertain. A previous report indicated that neoadjuvant therapy can improve survival by controlling regional extension [8]. Contrary to this, another report suggested that neoadjuvant chemotherapy decreased the survival rate compared to that on immediate resection [9]. Prospective studies are needed to establish neoadjuvant chemotherapy as a treatment for potentially resectable BTC.

PV cavernous transformation is the angiographic appearance of numerous collateral vessels around the PV owing to various reasons. These are classified as idiopathic causes (e.g., congenital malformation, or following hepatobiliary surgery) or secondary causes (e.g., liver cirrhosis, thrombosis, tumor) [10]. In this case, there were no hepatofugal collateral pathways caused by splenomegaly, a gastric or esophageal varix, or any hepatopetal collateral pathways. Furthermore, intraoperative findings suggested that the cavernous transformation was due to a congenital etiology, as the obstruction site was distant from the tumor and was soft under palpation. Unfortunately there is no previous CT or portography, however, it is speculated that the etiology of congenital PV cavernous transformation may result from portal vein malformation, or portal thrombosis for omphalitis or pylephlebitis.

Until October 2016, we experienced 15 cases of surgical resection for initially unresectable BTCs including this case. The periods of downsizing chemotherapy to surgical resection were varied; 22.7 ± 11.1 weeks (mean ± standard deviation). Among all 15 patients, N0 (no lymph node metastasis) was pathologically diagnosed for 9 patients and N1 (positive for lymph node metastasis) for 6 patients, and R0 resection was performed for 12 patients and R1 resection for 3 patients, respectively. According to Kaplan-Meier method, the MST of OS was calculated and log-lank test was used to test for significant differences between N0 and N1 group, and between R0 and R1 group. The MST of OS in N0 group (34.3 months) was significantly longer compared with that in N1 group (12.4 months) (*p* = 0.031). The MST of OS in R0 group was also significantly longer than that in R1 group (30.9 months for R0 group vs. 4.1 months for R1 group, *p* = 0.012). Although the number of cases is limited, these results implicated that N0 and R0 resection are important favorable prognostic factors for unresectable BTC patients who have undergone surgery after downsizing chemotherapy.

## Conclusions

We describe a case of successful surgical resection of initially unresectable ICC in a patient with PV cavernous transformation with suspected congenital origin. The combination of GEM and CDDP chemotherapy decreased tumor size and the viability of para-aortic lymph node metastases, enabling surgical resection to be performed. Downsizing chemotherapy with GEM plus CDDP may become an attractive treatment strategy for initially unresectable BTC. Further evidence and prospective studies are required to establish the optimal strategy for treatment of initially unresectable BTC.

## Abbreviations

BTC: Biliary tract cancer
CA19-9: Carbohydrate antigen 19-9
CDDP: Cisplatin
CT: Computed tomography
FDG: Fluorodeoxyglucose
GEM: Gemcitabine
ICC: Intrahepatic cholangiocarcinoma
IVC: Inferior vena cava
MRI: Magnetic resonance imaging
MST: Median survival time
OS: Overall survival
PET: Positron emission tomography
PV: Portal vein
